# Evaluation of Weight Gain, Clinicopathological and Radiographic Changes after Early Diagnosis and Treatment of Congenital Hypothyroidism in Cats

**DOI:** 10.3390/vetsci9030140

**Published:** 2022-03-16

**Authors:** Stefania Golinelli, Antonio Maria Tardo, Carla Giuditta Vecchiato, Eleonora Anicito Guido, Simone Perfetti, Alessia Diana, Federico Fracassi

**Affiliations:** Department of Veterinary Medical Sciences, University of Bologna, Ozzano dell’Emilia, 40064 Bologna, Italy; antoniomaria.tardo2@unibo.it (A.M.T.); carla.vecchiato2@unibo.it (C.G.V.); eleonora.anicito@gmail.com (E.A.G.); simone.perfetti4@unibo.it (S.P.); alessia.diana@unibo.it (A.D.); federico.fracassi@unibo.it (F.F.)

**Keywords:** feline congenital hypothyroidism, dwarfism, levothyroxine treatment, IGF-1

## Abstract

Congenital hypothyroidism is uncommon in cats. This case report describes weight gain, clinicopathological and radiographic changes after early diagnosis and treatment of congenital hypothyroidism in three British shorthair cats’ siblings. Data were assessed at 53 (diagnosis), 83, 185 and 365 days of age. Correlations between serum insulin-like growth factor-1 (IGF-1) and body weight, levothyroxine dose, total thyroxine, and thyroid-stimulating hormone concentrations were evaluated. The body weights of the congenital hypothyroid kittens were compared with those of their two healthy siblings and British shorthair kittens of the same age. At diagnosis, the congenital hypothyroid kittens showed a significantly lower body weight compared to the healthy siblings (*p* = 0.03). After diagnosis, oral levothyroxine supplementation was started. The difference in body weight was no longer observed after one month of treatment. The clinical signs, clinicopathological and radiographic abnormalities ameliorated after one month of treatment. IGF-1 concentration was significantly positively correlated with body weight (rs = 0.80, *p* < 0.002). In conclusion, resolution of the clinical signs, achieving a consistent within-breed weight, and improvement of the clinicopathological and radiographic parameters demonstrated the importance of the early diagnosis and treatment of feline congenital hypothyroidism.

## 1. Introduction

Congenital hypothyroidism (CH) is a relatively uncommon disease in cats, with about 70 cases reported in the literature [1,2,3]. It has been suggested that CH is an under-recognized disorder as a significative proportion of cases are not diagnosed or die at birth or when juvenile, without the cause of death being established [1,4,5,6]. All cases of CH in cats described so far have been diagnosed as primary hypothyroidism [1]. The latter can result from thyroid dysgenesis or, more commonly, from thyroid dyshormonogenesis [1]. Congenital central (pituitary or hypothalamic) hypothyroidism has not been well documented in cats [7,8]. Diagnosis of congenital hypothyroidism is based on clinical signs, supporting clinicopathology, radiographs, and thyroid function testing. The most reported clinical signs of CH are those exhibited as disproportionate dwarfism or cretinism. These signs include an enlarged broad head, a short neck and short limbs [1]. Additional findings are lethargy, mental dullness, constipation, hypothermia, bradycardia, prolonged retention of deciduous teeth, cold intolerance and retention of kitten hair, with the result that the coat remains soft and fluffy [1,3,4,5,6,7]. Clinicopathological abnormalities are non-specific and can include mild anemia, hypercholesterolemia, and azotemia [1]. The delayed appearance and closure of the ossification centers of the axial and appendicular skeleton are evident radiographically [1]. Other radiographic findings can include epiphyseal dysgenesis, reduced long bone length, shortened vertebral bodies, and scalloping of the ventral borders of the vertebral bodies, suggesting a lack of normal longitudinal growth [1]. These findings, associated with low-to-low normal total thyroxine (tT4) concentrations, together with high concentrations of endogenous thyroid-stimulating hormone (TSH), confirm primary hypothyroidism [9]. As hypothyroidism may be associated with abnormal liver function tests and low serum insulin-like growth factor 1 (IGF-1) concentrations, test results should be interpreted carefully when hepatic disorders or congenital hyposomatotropism are suspected [10]. Cats suffering from CH usually appear normal at birth; however, within the first 1–2 months of age, retarded growth becomes evident. CH should be considered as a differential diagnosis in kittens with failure to grow and/or clinical features of disproportionate dwarfism. A decrease in growth rate compared to littermates usually becomes evident by 6 to 8 weeks of age [1]. Since early diagnosis might bring a better prognosis, CH diagnosis should be confirmed by evaluating a complete serum thyroid profile which includes measurements of serum tT4 (+/− free T4 by dialysis) and TSH concentrations in all kittens with retarted growth [1]. The finding of low-to-low normal tT4 (with or without low free T4 by dialysis) concentrations together with a high concentration of TSH confirms primary hypothyroidism. It has been previously suggested that finding a high concentration of TSH is, probably, the single most important endocrine test for diagnosis of feline hypothyroidism. The main reasons for this postulate are two: (1) high concentrations have been reported in all of the congenitally hypothyroid cats previously described, and (2) falsely high values for TSH are not generally seen in cats with non-thyroidal illness [1]. Although the pathophysiological mechanisms, clinical aspects, diagnostic methods, and initial treatment for CH cats have been investigated in several reports [3,4,6,10,11,12,13,14,15,16,17,18,19,20,21,22,23,24,25], a complete long-term follow-up has rarely been described [3,6,10,16,25].This case series describes the body weight, clinicopathological and radiographic changes after early diagnosis, and the one-year treatment follow-up of three kittens with CH.

## 2. Material and Methods 

### 2.1. Sample Collection and Endocrine Tests

Blood specimens were collected by standard venipuncture using a blood vacuum collection system from three 53-day-old British shorthair (BS) kitten siblings, 2 entire females and 1 intact male presented to the Veterinary Teaching Hospital of the University of Bologna. Coagulated blood samples were centrifuged for 10 min at 3000× *g*; the serum was immediately transferred to plastic tubes, stored at 4 °C and analyzed the same day, or stored at −20 °C and thawed immediately before analysis. The blood samples for determining the complete blood count (CBC) were analyzed the same day as the sampling. The CBC was performed with an automated hematology analyzer (ADVIA 2120, Siemens Healthcare Diagnostics, Tarrytown NY, USA) while the chemistry parameters were measured using an automated chemistry analyzer (AU480, Beckman Coulter/Olympus, Brea, CA, USA).

Serum tT4, TSH, and IGF-1 were measured using a chemiluminescent enzyme immunoassay validated for use in cats [13,26]. The TSH stimulation test was carried out in one kitten by administering IV 75 μg of recombinant human TSH (rhTSH) and measuring the serum concentration of tT4 immediately before and 6 h after rhTSH administration [27]. 

### 2.2. Statistical Analysis 

The body weights (BWs) of the congenital hypothyroid kittens (CHk) were compared with those of the 2 healthy siblings (HS) and with the data recorded from 0, 53, 83, 185, and 365 day-old BS cats belonging to different BS breeders and private owners. Data from 0, 53, and 83 days of age were collected from 178, 95, and 42 kittens, respectively, these data coming from an Italian BS Breeder Association dataset. In addition, the BW values recorded at 185 and 365 days of age were collected from 28 and 26 BS cats, respectively, belonging to BS breeders as well as to private BS owners who answered a questionnaire. The Kruskal–Wallis test with the Dunn’s post-test was used for comparing BW among the 3 groups (CHk, HS, and BS kittens). Correlations between serum IGF-1 and BW, and tT4 and TSH concentrations, and levothyroxine dosage were evaluated, considering the data obtained at diagnosis and various follow-up examinations (83, 185, 365 days of age). In addition, the differences between serum IGF-1 concentrations at different time points (53, 83, 185, and 361 days of age) were compared using the Friedman test with Dunn’s post-test. The statistical analyses were carried out using GraphPad Prism version 9.2 (GraphPad Software, San Diego, CA, USA), with the significance level set at *p* < 0.05.

## 3. Ethical Consideration

This case report describes three client-owned cats admitted to the Veterinary Teaching Hospital of the University of Bologna for diagnosis and routine monitoring of CH. All diagnostic tests were performed as part of the work-up. No diagnostic tests or treatments were conducted for research purposes. The owners of each cat enrolled in the study signed an informed consent form.

## 4. Clinical Cases

### 4.1. Case Description

Three 53-day-old BS kitten siblings, 2 entire females (F1 = 0.62 kg BW; F2 = 0.50 kg BW) and 1 intact male (M1 = 0.60 kg BW), were presented to the Veterinary Teaching Hospital of the University of Bologna due to 2-week history of slower growth rate as compared to their normal littermates (1 intact male [0.86 kg BW] and 1 entire female [0.75 kg BW]). The owner also noted delayed tooth eruption and a decreased frequency of defecation. Moreover, the 3 kittens seemed to be both less active and playful than their littermates, and they were unable to learn new behavior (i.e. urinating in the litter box). The owner did not report previous medical problems. All the kittens lived with the mother cat and were nursed for the first 4 weeks of age. Starting from 4 weeks of age, the kittens had free access to a kitten wet food (Mother & Babycat, Royal Canin; Table S1) to be gradually consumed in larger amounts, with concomitant reduced consumption of the mother’s milk, and then, from 6 weeks of age, they were fed a kitten dry diet (Kitten, Royal Canin; Table S1.). 

**Figure 1:**
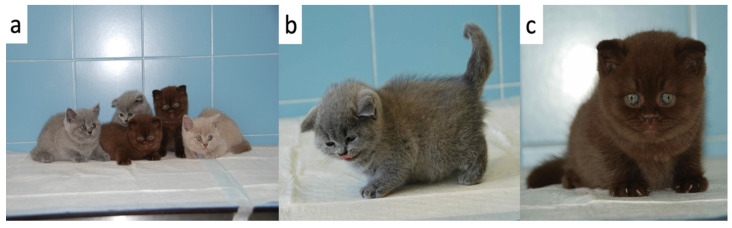
Congenital hypothyroid kittens and healthy siblings (**a**) The 3 kittens with growth failure (in the middle) together with their healthy littermates (left and right) at the time of presentation (**b**) One of the 3 kittens (kitten F1) with a short block-like body and a fluffy hair coat (**c**) One of the 3 kittens (kitten M1) with a short neck, blue eyes, and ventral curling of the pinnae.

**Figure 2:**
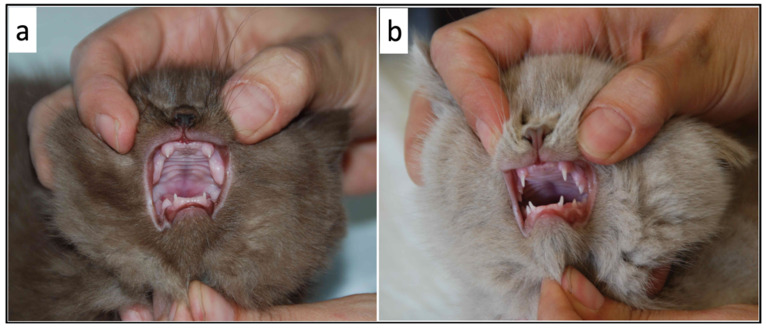
Comparison of tooth eruption between two siblings (**a**) Delayed tooth eruption in one of the 3 kittens with growth failure (**b**) Normal tooth eruption in one of the healthy littermates.

Compared to their littermates, at physical examination, the 3 kittens were lethargic, dull, and smaller having a disproportionate appearance; the body condition score was 5/9 (according to Laflamme 9-point scale modified by Ghielmetti et al. 2017) [28,29]. In particular, a broad head, short neck and limbs, and a short block-like body were evident in the 3 kittens (Figure 1). Moreover, they showed a soft and fluffy hair coat, ventral curling of the pinnae and delayed tooth eruption (Figure 2). Additional findings were mild hypothermia (37.7–37.8 °C) and, in 1 of the kittens, slight distension of the colon with the presence of firm stools on abdominal palpation. The remainder of the physical examination was unremarkable. Palpation of the thyroid gland did not reveal any abnormalities.

Hematology and serum biochemistry, evaluated just in 1 kitten (F1), due to an insufficient blood sample, revealed some non-specific age-related findings, such as low hematocrit (26.9%; reference range [RR] 32–48%) and erythrocyte count (6.01 × 106/µL; RR 7–11 × 106/µL), and decreased total protein concentration (5.35 g/dL; RR 6.5–8.8 g/dL). In addition, increased cholesterol was detected (307 mg/dL; RR 59–230 mg/dL). 

**Figure 3:**
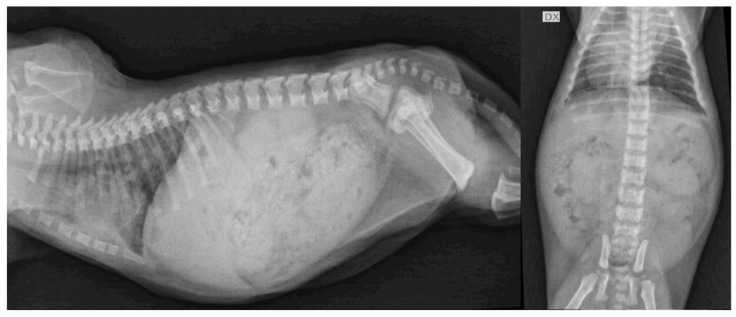
Lateral and ventrodorsal projections of thorax and abdomen of 1 hypothyroid kitten (F2) at the time of diagnosis. Delayed appearance of ossification centers with reduced long bone length and shortened vertebral bodies with scalloped ventral borders were observed. Right-sided displacement of the cardiac apex likely compatible with sternal malformation was also noted. Moderate distension of the gastrointestinal tract was also present. DX, right side.

Radiographic examination revealed shortened vertebral bodies, no evidence of growth plate, and epiphyseal dysgenesis of the long bones in all three kittens (Figure 3). Moderate distension of the colon was observed in one of the kittens (F2). 

At this stage, CH, congenital hyposomatotropism and mucopolysaccharidosis were considered to be the most likely differential diagnoses. 

To evaluate the possible presence of hypothyroidism, serum tT4 and TSH were measured. In addition, a TSH stimulation test was carried out in 1 kitten. The thyroid hormones of the 3 kittens are reported in Table 1. In 1 cat, there was not enough serum to measure the tT4.

The IGF-1 concentration was measured in all 3 kittens in order to investigate hyposomatotropism and was found to be decreased in all 3 kittens (Table 2). The results of the serum tT4/TSH concentrations, the TSH stimulation test and the radiographic findings were indicative of hypothyroidism and treatment with oral solution levothyroxine supplementation (Leventa, MSD, Boxmeer, The Netherlands); 20 mcg/kg q24h was initiated [1]. The overall behavior of the kittens and the frequency of defecation ameliorated within a few days after starting therapy. After one month, the kittens were found to be more alert, active, and playful. In addition, tooth eruption markedly improved. Serum tT4 and TSH concentrations were measured 12 h after the evening levothyroxine dose [1]. Serum tT4 values were within normal limits; however, increased TSH concentrations persisted in two kittens (Table 3). 

**Figure 4:**
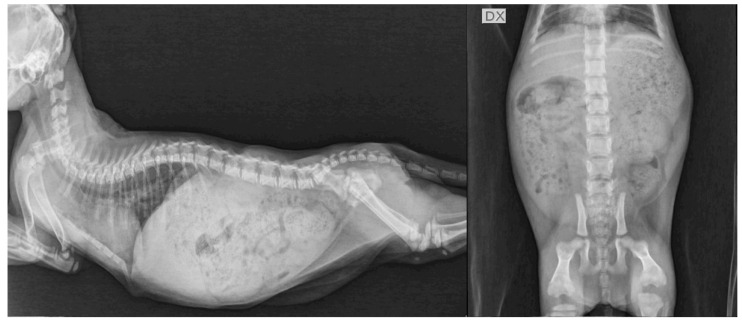
Lateral projection of thorax and abdomen and ventro-dorsal projection of the abdomen the same hypothyroid kitten (F2) of Figure 3 at 83 days of age (after 1 month of levothyroxine treatment). Normal appearance of growth centers of long bones and vertebral bodies are visible. Moderate distension of the gastrointestinal tract was also present. DX, right side.

Moreover, at the first re-evaluation at 83 days of age, the IGF-1 concentration (Table 2) increased to a normal value in 2 of 3 kittens in correspondence with an improvement in radiographic abnormalities (Figure 4).

**Figure 5:**
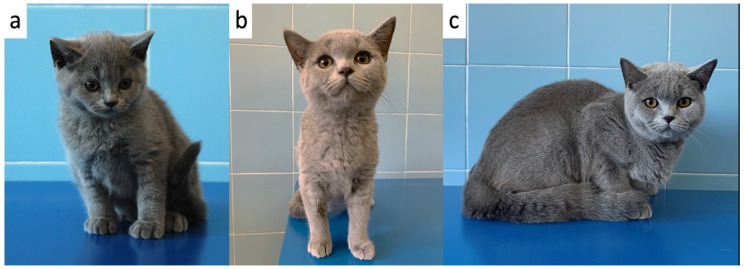
One of the 3 kittens (kitten F1) at 83 (**a**), 185 (**b**) and 365 days of age (**c**).

The levothyroxine dose (20 μg/kg q24h) was maintained unchanged at the first re-evaluation in all 3 kittens while the amount of levothyroxine supplementation was adapted to the increasing body weight. The kittens were re-evaluated several times during the following months, and the levothyroxine dosage was adjusted according to tT4/TSH concentrations (Table 2) and increasing BW. At the time of writing (365 days of age, 10 months after initiating therapy), the kittens are doing well (Figure 5), and the serum concentrations of tT4 and TSH had normalized (last levothyroxine dose: 10 μg/kg [F1], 18 μg/kg [F2], 20 μg/kg [M1]). 

### 4.2. Body Weight Changes and IGF-1 Concentrations

**Figure 6:**
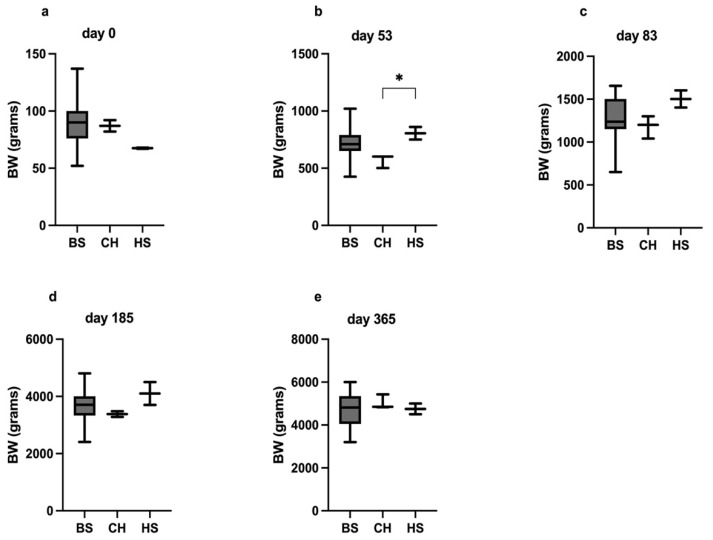
The differences in BW among the groups at days 0 (**a**), 53 (**b**), 83 (**c**), 185 (**d**), and 365 (**e**). The * indicates a significant difference in the multiple comparisons Dunn’s test, *p* < 0.05.

At birth, no differences in BW (median [g] and range) were observed among the CHk (87, 82–92), HS (67, 61–91), and BS (90, 52–137) kittens (*p* = 0.11; Figure 6). At diagnosis (53 days of age), the CHk had a significantly lower BW as compared to their HS (600 [500–600] vs. 805 [750–860], *p* = 0.03). Moreover, at 53 days of age, the BW of the CHk tended to be lower, although not significantly lower than that of the BS kittens (600 [500–600] vs. 710 [426–1020]; *p* = 0.06). The difference in BW between the CHk (1200, 1040–1300) and the HS (1500, 1400–1600) was no longer observed at 83 days of age after 1 month of levothyroxine treatment (*p* = 0.26). 

**Figure 7:**
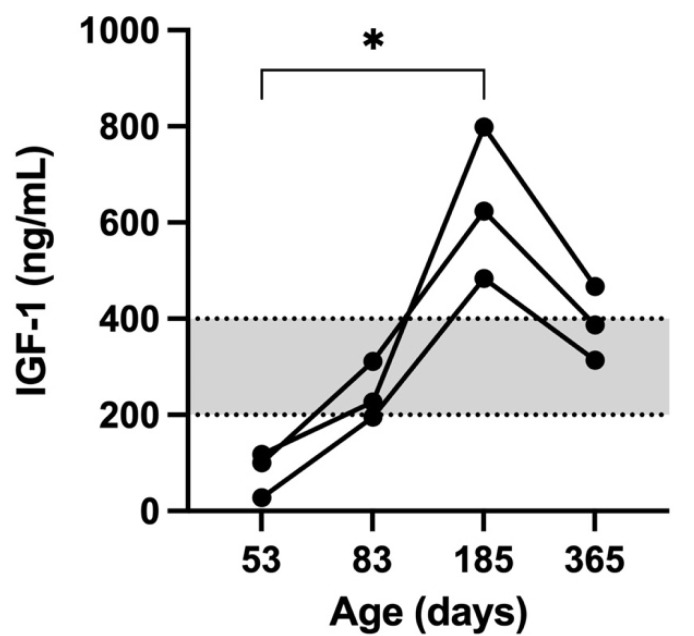
The IGF-1 concentrations (ng/mL) in the 3 kittens at 53, 83, 185, and 365 days of age. The grey area represents the reference range for IGF-1. The * indicates a significant difference (*p* < 0.05) in IGF-1 concentrations between 53 and 185 days of age.

A significant positive correlation was detected only between IGF-1 concentration and BW (rs = 0.80, *p* < 0.002) while no correlation was observed between IGF-1 and tT4, TSH and levothyroxine dosage. The IGF-1 concentration was significantly higher at day 185 when compared to day 53 (*p* = 0.002; Figure 7). 

## 5. Discussion

The present study describes a one-year follow-up of three kittens with an early diagnosis of CH. The clinical signs of the kittens were highly suggestive of hypothyroidism and consistent with those observed in previous reports of feline CH, even if a palpable goiter was not present. A goiter is expected in congenital hypothyroidism due to dyshormonogenesis; however, it may not be apparent until 6 months of age (the time needed for high-circulating TSH to stimulate gross thyroid hyperplasia) [1]. The diagnosis of hypothyroidism was supported by the radiographic and clinicopathological changes and was confirmed by the results of basal thyroid hormone analyses (i.e. serum tT4 and/or TSH concentrations). In the present report, the owner was very observant in identifying the early clinical signs; this allowed an earlier diagnosis of the disease in comparison with that reported in the literature (53 days vs. 98, 84, and 196 days) [4,6,10]. The serum IGF-1 concentrations were measured to investigate hyposomatotropism and low results were found in all three kittens. Low IGF-1 concentration has already been described in a cat with CH [10]. Several explanations for the low IGF-1 result were postulated by the authors: (1) presence of concurrent hyposomatotropism and hypothyroidism, (2) it could be influenced by the young age of the kitten, (3) it could be a consequence of reduced liver function, and (4) it could be a result of hypothyroidism. Taking into account the clinical signs, in addition to the clinicopathological and radiographic changes of the three kittens, the latter explanation was considered the most likely. As a matter of fact, it has been previously described in humans, dogs, and rats how the thyroid status can influence the growth hormone axis and IGF-1 concentration has been shown to increase in hypothyroid humans after thyroxine replacement therapy [30,31]. One month after initiating levothyroxine therapy, indeed, the IGF-1 concentration normalized in two of the three kittens; therefore, it was unlikely that concurrent hyposomatotropism could have been a reason for the initial IGF-1 low concentration. For this reason, the result of low IGF-1 concentrations in cats with CH should be interpreted carefully. 

All the kittens had a good response to therapy with oral levothyroxine, and the clinical signs ameliorated within one month of therapy, as previously reported in the literature [3,10,24]. The kittens were re-evaluated several times during the following months, and the levothyroxine dosage was adjusted according to their tT4 concentrations and body weight changes. Even though it was possible to improve the clinical signs and maintain near-normal concentrations of tT4 after the initiation of therapy, normalization of the TSH concentrations was challenging. It was assumed this indicated a partial deficiency of thyroid hormones (i.e. subclinical hypothyroidism) and the need, especially in the early stages of the condition, to adjust the levothyroxine dose more frequently in relation to the body weight [32]. In the present study, a normal TSH concentration was obtained in all the cats after 10 months of therapy. This was in contrast with a previous study in which the TSH normalized after 8–10 weeks of levothyroxine treatment [3]. The discrepancy between studies may be due to the fact that, in the study of Iturriaga et al. [3], cats of different ages (range 4–19 months) were included. These cats were older than the ones reported herein; therefore, the effect of growth could reasonably have a lower impact on levothyroxine dosage adjustments. Radiographic features were strongly suggestive of hypothyroidism and similar to those reported in the literature [24,29,32]. The main radiographic findings observed in the present study were delayed epiphyseal ossification with no evidence of growth plates in the appendicular and axial skeleton. As a consequence, reduced long bone length and a shortened vertebral body were noted. Those signs are not pathognomonic for CH because they have been described in cats affected by epiphyseal dysplasia or panhypopituitarism [24,33,34]. Therefore, a clinicopathological diagnosis was needed. Other radiographic changes reported in cats affected by hypothyroidism are valgus deformities of the appendicular skeleton due to retarded ossification and secondary osteoarthrosis [24,33,34]. Those signs were not observed in our kitten, probably due to the early diagnosis and treatment of hypothyroidism. In all the patients, the radiographic examination performed after one month of therapy showed the appearance of growth plates in the axial and appendicular skeleton and no other abnormalities were detected. Little information regarding radiographic follow-up in hypothyroid cats is present in the literature [24,33,34].

The importance of an early diagnosis and consequent better prognosis highlighted as CH should be considered a possible differential diagnosis in kittens with retarded growth and/or clinical features of disproportionate dwarfism. The finding of low-to-low normal tT4 concentrations together with a high concentration of TSH confirms the diagnosis of primary hypothyroidism and leads to the start of the levothyroxine treatment.

In order to assess the relationship between the IGF-1 concentration and the weight gain during the growing phase of the kittens, leftover samples were used to measure the IGF-1 at each follow-up examination. The plasma concentration of IGF-1 is regulated by the growth hormone (GH) which stimulates IGF-1 synthesis in the liver and other tissues; IGF-1 is involved in the growth and function of almost every organ in the body [35,36]. Thyroid status influences the growth hormone axis in humans, dogs, cats, and rats, and IGF-1 concentration has been shown to increase in hypothyroid humans after levothyroxine therapy [10,30,31].

A clear correlation between body weight and IGF-1 concentration in growing cats has never been described. In the present study, a strong positive correlation was found between IGF-1 concentration and BW. It has been previously reported that IGF-1 concentrations are higher during the growing phase in comparison with the adult phase in both humans and cats [37,38,39]. The present results agreed with a previous study in which the IGF-1 concentration in growing cats, followed until 21 months of age, reached its highest value at 5 months [37]. In this study, the highest concentration of IGF-1 was detected in the kittens at 185 days of age; in addition, a significant difference was found between the IGF-1 concentration measured at 53 and 185 days. Moreover, the IGF-1 concentration decreased at the last follow-up (365 days), although the difference was not significant.

The IGF-1 trend pointed out in this case report is similar to that described in children.

It has been reported that the serum IGF-1 levels tend to increase slowly with age in childhood, reaching a peak level at puberty, and decreasing with age thereafter [40,41]. The same developmental pattern of serum IGF-1 was present in all three kittens with CH. As in humans, the kittens of this study also showed the IGF-1 peak level at the age of sexual maturation (185 days) followed by a decreased concentration at 1 year of age. These high IGF-I concentrations in humans are likely explained as the result of the sex hormone-induced increasing GH secretion in the pubertal years [42]. However, a direct action of sex steroids on IGF-I levels cannot be excluded [43]. The same mechanism could explain the IGF-1 pattern in our kittens and it has already been previously described in a previous study [39].

In the present study, no correlation between IGF-1 concentration and levothyroxine dosage or thyroid hormones (tT4 and TSH) was observed. The discrepancy between the present findings and those reported in human medicine [30,31] may be due to the small sample size and differences between species. 

To the authors’ knowledge, the BW gain and the comparison of BW data with those from healthy kittens have never previously been described in CHk. The CHk in the present study showed a significant BW difference at diagnosis (53 days) in comparison with the HS. As previously reported, affected kittens typically appear normal at birth; however, noticeable changes become evident by 6 to 8 weeks of age, as described in the CHk in this study [1].

This report warrants to highlight how the differences in BW of the CHk, and the HS and BS kittens were no longer identified after a month of treatment. The time required for the BW to improve is therefore similar to that needed by clinical, clinicopathological, and radiographic abnormalities to improve after the beginning of CH treatment [3,10,24].

The first limitation of this study is the low number of patients included. Secondly, due to the small size of the kittens and the need for a large sample of blood, T4 was not measured in one of the kittens at the time of diagnosis. This may have partially influenced our results.

In summary, findings in these cases indicate the importance of the early diagnosis and treatment of feline CH in order to obtain a complete resolution of the clinical signs, together with reaching a consistent within-breed BW, and improvement of the clinicopathological and radiographic parameters. Overall, in this report, the authors speculated that, in addition to thyroid status, IGF-1 concentrations and their trend in CHk should be interpreted carefully owing to the influence of the growth phase. 

## Figures and Tables

**Table 1:** Serum tT4 and TSH concentrations, and TSH stimulation test results (0 h and 6 h) at diagnosis.

Parameter	Kitten F1	Kitten F2	Kitten M1	Reference Range
Baseline tT4 (nmol/L)	--	12.9	6.44	15–42
tT4 after rhTSH (nmol/L)	--	12.9	--	15–42
TSH (ng/mL)	5.44	6.20	12.00	0.03–0.30

tT4, total thyroxine; rhTSH, recombinant human thyroid-stimulating hormone TSH; TSH, thyroid-stimulating hormone; F1, female 1; F2, female 2; M1, male 1.

**Table 2:** The IGF-1 concentrations at diagnosis (53 days) and during the follow-up examinations.

IGF-1 (ng/mL)	53 Days	83 Days	185 Days	365 Days	Reference Range
Kitten F1	100.60	311.50	623.40	387.30	200–400
Kitten F2	27.51	195.50	483.70	313.80	200–400
Kitten M1	117.50	227	798.50	467.10	200–400

IGF-1, insulin-like growth factor 1; F1, female 1; F2, female 2; M1, male 1.

**Table 3:** Serum tT4 and TSH concentrations during the follow-up examinations.

Kittens	Parameter	83 Days	185 Days	365 Days	Reference Range
Kitten F1	tT4 (nmol/L)	63.40	45.40	31.8	15–42
	TSH (ng/mL)	0.26	0.05	0.03	0.03–0.30
Kitten F2	tT4 (nmol/L)	34.40	15.20	21.6	15–42
	TSH (ng/mL)	1.00	7.57	0.15	0.03–0.30
Kitten M1	tT4 (nmol/L)	45	28.20	46.2	15–42
	TSH (ng/mL)	0.77	2.81	0.07	0.03–0.30

tT4, total thyroxine; TSH, endogenous thyroid-stimulating hormone; F1, female 1; F2, female 2; M1, male 1.

## Data Availability

The data presented in this study are available in the manuscript.

## Supplementary Materials

The following are available online at https://www.mdpi.com/article/10.3390/vetsci9030140/s1. Table S1: Average composition of the diets fed to kittens.
