# Supplementary material for: Evaluation of Weight Gain, Clinicopathological and Radiographic Changes after Early Diagnosis and Treatment of Congenital Hypothyroidism in Cats
Source: Vet Sci. 2022 Mar 16;9(3):140. doi: 10.3390/vetsci9030140 (PMC8950234; doi:10.3390/vetsci9030140)
Supplement: Supplementary file 1 [file vetsci-09-00140-s001.zip › vetsci-1615438-supplementary.pdf]

veterinary sciences

MDPI

Evaluation of Weight Gain, Clinicopathological and Radiographic Changes after early Diagnosis and Treatment of Congenital Hypothyroidism in Cats

Stefania Golinelli *,†, Antonio Maria Tardo †, Carla Giuditta Vecchiato, Eleonora Anicito Guido, Simone Perfetti, Alessia Diana and Federico Fracassi

Supplementary File 1

Table S1. Average composition of the diets fed to kittens. Data are expressed on as fed basis (%).

	Wet food[a]	Dry food[b]
Moisture	79	8
CP	10.5	36
EE	5.5	18
Ash	1.9	7.7
CF	0.9	2.3
Metabolizable Energy (kcal ME/kg)[c]	1040	4087

CF: crude fiber; CP: crude protein; EE: ether extract.

[a]First Age Mother & Babycat, Royal Canin. Ingredient list: Water sufficient for processing, chicken liver, chicken, chicken by-products, pork by-products, wheat gluten, powdered cellulose, modified corn starch, pork plasma, natural flavors, vegetable oil, brewers rice flour, fish oil, hydrolyzed yeast, calcium carbonate, carrageenan, sodium tripolyphosphate, guar gum, potassium phosphate, taurine, vitamins [DL-alpha tocopherol acetate (source of vitamin E), L-ascorbyl-2-polyphosphate (source of vitamin C), thiamine mononitrate (vitamin B1), niacin supplement, biotin, D-calcium pantothenate, riboflavin supplement, pyridoxine hydrochloride (vitamin B6), folic acid, vitamin B12 supplement, vitamin D3 supplement], choline chloride, potassium chloride, trace minerals [zinc proteinate, zinc oxide, ferrous sulfate, copper sulfate, manganous oxide, sodium selenite, calcium iodate], marigold extract (Tagetes erecta L.), magnesium oxide, carotene.

[b]Second Age Kitten, Royal Canin. Ingredient list: Chicken by-product meal, brown rice, corn gluten meal, brewers rice, chicken fat, wheat gluten, corn, natural flavors, egg product, dried plain beet pulp, powdered cellulose, fish oil, monocalcium phosphate, calcium carbonate, grain distillers dried yeast, vegetable oil, sodium silico aluminate, potassium chloride, psyllium seed husk, fructooligosaccharides, salt, choline chloride, vitamins [DL-alpha tocopherol acetate (source of vitamin E), L-ascorbyl-2-polyphosphate (source of vitamin C), niacin supplement, biotin, riboflavin supplement, D-calcium pantothenate, pyridoxine hydrochloride (vitamin B6), vitamin A acetate, thiamine mononitrate (vitamin B1), vitamin B12 supplement, folic acid, vitamin D3 supplement], L-lysine, sodium pyrophosphate, hydrolyzed yeast, DL-methionine, taurine, trace minerals [zinc proteinate, zinc oxide, ferrous sulfate, manganese proteinate, manganous oxide, copper sulfate, calcium iodate, copper proteinate, sodium selenite], marigold extract (Tagetes erecta L.), magnesium oxide, rosemary extract, preserved with mixed tocopherols and citric acid.

[c] Metabolizable Energy (ME) calculated according NRC (2006).

Voce bibliografica da aggiungere:

NRC - National Research Council (2006). "Energy," in Nutrient requirements of dogs and cats, ed. National Academies Press (Washington, DC, USA), 28–48.
